# Self-efficacy and application of skills in the workplace after multidisciplinary trauma masterclass participation: a mixed methods survey and interview study

**DOI:** 10.1007/s00068-022-02159-8

**Published:** 2022-11-10

**Authors:** Frederike J. C. Haverkamp, Idris Rahim, Rigo Hoencamp, Cornelia R. M. G. Fluit, Kees J. H. M. Van Laarhoven, Edward C. T. H. Tan

**Affiliations:** 1 Department of Surgery (Internal Postal Code 618), Radboudumc, P.O. Box 9101, 6500 HB Nijmegen, The Netherlands; 2 Department of Surgery, Alrijne Medical Centre, Leiderdorp, The Netherlands; 3 Department of Surgery, Erasmus Medical Center, Rotterdam, The Netherlands; 4 Defence Healthcare Organization, Ministry of Defence, The Hague, The Netherlands; 5 Department of Surgery, Leiden University Medical Centre, Leiden, The Netherlands; 6 Radboudumc Health Academy, Nijmegen, The Netherlands

**Keywords:** Team training, Damage control surgery, Trauma masterclass

## Abstract

**Purpose:**

The most complex injuries are usually least often encountered by trauma team members, limiting learning opportunities at work. Identifying teaching formats that enhance trauma skills can guide future curricula. This study evaluates self-assessed technical and nontechnical trauma skills and their integration into novel work situations for multidisciplinary trauma masterclass participants.

**Methods:**

This mixed methods study included participants of a multidisciplinary 3-day trauma masterclass. Ratings of trauma skills were collected through pre- and postcourse questionnaires with 1-year follow-up. Qualitative semi-structured interviews 9 months postcourse focused on the course format and self-perceived association with technical and nontechnical skills applied at work.

**Results:**

Response rates of pre- and postcourse questionnaires after 1 day, 3 months, and 1 year were respectively 72% (51/71), 85% (60/71), 34% (24/71), and 14% (10/71). Respondents were surgeons (58%), anesthesiologists (31%), and scrub nurses (11%). Self-efficacy in nontechnical (mean 3.4, SD 0.6 vs. mean 3.8, SD 0.5) and technical (mean 2.9, SD 0.6 vs. mean 3.6, SD 0.6) skills significantly increased postcourse (*n* = 40, *p* < 0.001). Qualitative interviews (*n* = 11) demonstrated that increased self-efficacy in trauma skills was the greatest benefit experienced at work. Innovative application of skills and enhanced reflection demonstrate adaptive expertise. Small-group case discussions and the operative porcine laboratory were considered the most educational working formats. The experienced faculty and unique focus on multidisciplinary teamwork were highly valued.

**Conclusion:**

Course participants’ self-assessed work performance mostly benefited from greater self-efficacy and nontechnical skills. Future trauma curricula should consider aligning the teaching strategies accordingly.

**Supplementary Information:**

The online version contains supplementary material available at 10.1007/s00068-022-02159-8.

## Introduction

The most complex injuries trauma care providers encounter are often those most infrequently covered during the training years. For example, penetrating trauma can result in severe injuries requiring prompt management, whereas its incidence is under 4% in various developed countries worldwide [1–4]. When occasionally encountering severe, complex injuries, teaching possibilities in the workplace remain limited because of time pressure and the fact that there is a life at stake. A feeling of insufficient preparedness for patient care can seriously burden healthcare providers, as exemplified by the COVID-19 pandemic [5]. Additionally, inadequate preparedness could result in inferior patient outcomes.

Especially in teams with limited exposure, preparedness to handle uncommon complex trauma cases does not solely require sufficient domain-specific and technical skills, but also nontechnical skills, such as strong communication, teamwork, and adaptivity [6]. The corresponding self-efficacy is equally important for healthcare providers to feel confident enough to apply their skills [7].

Various courses have been developed to provide alternative training opportunities in trauma care, beyond the basic principles of the Advanced Trauma Life Support (ATLS). The Advanced Surgical Skills for Exposure in Trauma (ASSET), Advanced Trauma Operative Management (ATOM), Definitive Surgical and Anesthetic Trauma Care (DSTC and DATC), and Norwegian trauma courses have demonstrated a correlation with greater trauma skills and self-efficacy levels [8–13]. However, the educational rationale on how these teaching formats enhance domain-specific skills and self-efficacy in the workplace has been insufficiently explored.

Furthermore, the extent to which current trauma courses contribute to adaptivity has not been assessed, whereas this skill is essential for trauma care. In the extremely fast-evolving healthcare field, adaptivity facilitates problem-solving even in rarely encountered, highly complex injuries that trauma care providers are confronted with. Great adaptivity is reflected in a construct called adaptive expertise, which entails the ability to overcome changes in work requirements using expert knowledge in innovative ways [14–16].

Despite previous efforts to guide medical education to promote adaptive expertise [17, 18], current (post)academic teaching efforts in this field remain limited or insufficient [19, 20]. Work experience itself is not associated with higher levels of adaptive expertise, indicating that explicit learning efforts are required [15, 21]. It is hypothesized that teaching formats that enhance self-efficacy might foster adaptive expertise, as a positive association has been demonstrated between self-efficacy and innovative behavior in the workplace [22–24].

Insight into the educational value of the currently applied teaching formats regarding their stimulation of technical and nontechnical trauma skills can guide future trauma care curricula. Therefore, this study aims to evaluate trauma care providers’ self-assessed technical and nontechnical trauma skills and gain a deeper understanding of their integration into novel work situations after participation in the Dutch Definitive Surgical and Anesthetic Trauma Care (DSTC and DATC) course, a multidisciplinary trauma masterclass.

## Methods

This mixed methods study consists of quantitative longitudinal digital questionnaires and qualitative semi-structured interviews. The Checklist for Reporting Of Survey Studies and Standards for Reporting Qualitative Research guidelines were used to ensure proper reporting of methods, results, and discussion of the self-efficacy survey and semi-structured interviews, respectively.

### Setting

The Definitive Surgical and Anaesthetic Trauma Care (DSTC and DATC) courses are organized worldwide. The Dutch DSTC and DATC courses are combined into a three-day multidisciplinary masterclass (the DSATC course) [25]. The multidisciplinary aspect established by integrating the surgical and anesthetic courses is a unique feature implemented by the Dutch organizational committee.

The DSATC course of 2019 consisted of individual theoretical preparation (DSTC course manual and recommended articles on anesthesiology) followed by three physical course days filled with lectures, small-group case discussions, and case-based hands-on workshops in the anatomy and operative porcine laboratories. In the anatomy laboratory, emergent exposure of vital structures, vascular control, abdominal and pelvic packing, and external fixator placement are practiced on cadavers.

In the operative porcine laboratory, DATC course participants will practice anesthesia skills simultaneously with DSTC course participants who practice their surgical skills. Each operating table generally functions with two surgery participants, one anesthesiology participant, one anesthesia nurse, one scrub nurse, and one anesthesiology and one surgery faculty member. Participants get presented with background information on a patient case on which they base their approach. Faculty members are the only people in the operation room who are aware of where injuries are located beforehand, while course participants have to locate, expose and treat the injuries as quickly as possible. Injuries included in this workshop are those to the heart, lung, liver, spleen, digestive tract, kidney, bladder, or vascular injuries including laceration of the vena cava inferior. Interdisciplinary collaboration and practice of communication skills are highly integrated into all working formats, but especially within the operative porcine laboratory.

DSATC course faculty are highly experienced specialists in trauma care. Before receiving accreditation as an official faculty member, these specialists have to participate in a teach-the-teacher course. They have to attend the DSATC course as ‘faculty potential’, meaning their educational skills are supervised and assessed by senior faculty members and discussed at the faculty meeting.

### Participants

The study population comprised senior residents or attending physicians in trauma surgery (including orthopedic surgeons) or anesthesiology and scrub nurses who participated in the Dutch DSATC course in November 2019 (*n* = 71).

### Data collection and analysis

#### Quantitative questionnaire

Invitations for participation in this digital online questionnaire were sent by email together with a unique invitation link at four time points: precourse questionnaires 1 day prior to the DSATC and postcourse questionnaires after 1 day, 3 months, and 1 year. DSATC participants were reminded during the course and additional reminders for postcourse questionnaires were sent four weeks after the initial invitation.

Background characteristics were collected together with quantitative ratings of the participants’ confidence in various technical (e.g., performing a surgical airway) and nontechnical (e.g., communication, leadership, and teamwork) skills measured on a 5-point Liker scale (1 fully incompetent – 5 fully competent), see Online Resource 1 DSATC self-efficacy questionnaire. The included skill items were based on course content, expert opinion, and a previously conducted similar questionnaire [8, 10]. Other than expert review by trauma and educational specialists, and its previous application, this questionnaire was not further validated [8, 10].

Data were collected anonymously and stored on encrypted servers. After respondents were informed about the study goal, design, data protection and the voluntary nature of their participation, they consented to participate by submitting their responses.

Descriptive statistics are displayed as numbers with percentages or the mean with the standard deviation. All variables concerning skill ratings were categorized into nontechnical, technical, and bleeding management skills with pooled ratings per category. Bleeding management requires a combination of technical skills and domain-specific knowledge and is therefore categorized separately. The primary outcome measure was the rating of self-assessed skills per pooled category. Precourse ratings were compared with the postcourse ratings after one day using the Wilcoxon signed-rank test. Although the Wilcoxon signed-rank test is a nonparametric test, the included values are reported as the mean with the standard deviation instead of the median with the interquartile range because the means provided more detailed insight into the observed differences, and no substantial outliers were observed that needed correction using the medians. Results from the follow-up questionnaire after three months and one year were not included in the comparative analyses due to the low absolute number of respondents.

All statistical analyses were performed using IBM SPSS Statistics v. 25. An *α* level of 0.05 or lower was regarded as significant. Missing data were considered missing at random and were dealt with accordingly by excluding missing variables per analysis. The Wilcoxon signed-rank test excluded unpaired cases from the analysis.

Dropouts were compared with respondents included in the follow-up. Due to the low number of dropouts (*n* = 8), this comparison was not performed using statistical analyses but with inspection of each dropout to identify potential outliers.

### Semi-structured interviews

Interviews were held during August 2020, 9 months after the DSATC course participation. For the selection of participants, purposive sampling was performed based on profession and work experience to compose a representative study population. Over 75% of the course participants are trauma surgeons, orthopedic surgeons, anesthesiologists, and corresponding residents. Thus, the interviews focused on these professions and excluded scrub or anesthesia nurses.

Study participants were contacted by email to schedule the interview, and a reminder was sent after 1 week. Participants were recruited until data saturation was reached, meaning that no new insight was gathered during the last interviews. The telephonic interviews (due to the COVID pandemic) were conducted in Dutch by one researcher (I.R.) and lasted approximately 30 min. Before each interview, subjects were asked whether they were fully informed about the research scope and provided informed consent for participation.

The interview focused on learning activities related to course participation and its experienced association with changes in technical and nontechnical skills applied at work. The interview guide (Online Resource 2 Interview guide) was based on the course content and expert opinions of education and trauma care specialists. Questions related to adaptive expertise as a nontechnical skill were partly based on a study by Bohle et al., who developed a measurement tool for adaptive expertise called the Adaptive Expertise Inventory [21]. This tool was developed primarily in the context of professional, scientific, and technical areas, rather than the human health sector. Therefore, adjustments were made based on consultation with an experienced educational specialist and author. A pilot interview with a senior DSATC faculty member and author resulted in minor structural modifications.

Interviews were recorded using Audacity software v. 2.4.2 and stored on encrypted servers. Subsequently, interviews were transcribed in Dutch using F4 software v. 7, and the transcriptions were anonymized. Two investigators (F.H. and I.R.) coded the transcriptions using a combined inductive and deductive approach using Atlas.TI software. The deductive approach means that attention was paid to identify statements related to specific topics of interest, such as the development of adaptive expertise and how newly acquired skills are transferred to the work environment. Other codes (those correlating to the themes of personal context and learning context, see “Results” section) were derived using the inductive approach.

Consecutively, the thematic analysis was performed through a constant comparative approach, meaning that multiple codes covering the same topic were categorized into themes and were continuously compared to previously coded data. The interview guide and illustrative quotes were translated to English for this manuscript. Initial analysis of the interviews was performed by two researchers (F.H. and I.R.) and data interpretation by all researchers to limit potential bias that could have resulted if the sole interviewer (I.R.) might be able to recall participants’ identities when reading the transcripts.

### Ethical considerations

A Medical Ethics Committee deemed this study exempt from the Medical Research Involving Human Subjects Act (No. 2020-6771). Online Resource 3 contains a reflexivity statement.

## Results

### Quantitative questionnaire

Response rates at each measurement point were 72% (51/71) precourse, 85% (60/71) postcourse after 1 day, 34% (24/71) postcourse after 3 months, and 14% (10/71) postcourse after 1 year. There were 40 paired responses of participants who completed the pre- and one-day postcourse questionnaires. Respondents' background characteristics are listed in Table 1. Background characteristics and precourse skill ratings of dropouts (*n* = 8) did not substantially differ from respondents included in the follow-up (Online Resource 4 Characteristics of dropouts).

**Table 1:** Background characteristics of respondents

Characteristic	Value (*n* = 71)
Sex (*n*, %)	
Male	51 (72%)
Female	20 (28%)
Age (mean, SD)	37.5 (SD 6.1)
Profession (*n*, %)	
Surgeon	41 (58%)
Scrub nurse	8 (11%)
Anesthesiologist	22 (31%)
Trauma center level^a^ of current worksite (*n*, %)	
Level 1	38 (54%)
Level 2	21 (30%)
Level 3	7 (10%)
Other	5 (7%)
Years of experience in trauma care (mean, SD)	5.7 (SD 4.7)
Military experience (*n*, %)	
Yes	8 (11%)
No	63 (89%)
Experience working in austere environment (*n*, %)	
Yes	11 (16%)
No	60 (85%)
Number of patients treated each year with traumatic injury (*n*, %)	
0	1 (1%)
1–4	5 (7%)
5–9	5 (7%)
10–14	7 (10%)
≥ 15	53 (75%)
Number of patients treated each year with ISS > 15 (*n*, %)	
0	10 (14%)
1–4	21 (30%)
5–9	17 (24%)
10–14	6 (9%)
≥ 15	17 (24%)
Number of patients treated each year with penetrating trauma (*n*, %)	
0	19 (27%)
1–4	37 (52%)
5–9	9 (13%)
10–14	4 (6%)
≥ 15	2 (3%)
Number of patients treated each year requiring damage control surgery (*n*, %)	
0	21 (30%)
1–4	34 (48%)
5–9	7 (10%)
10–14	3 (4%)
≥ 15	6 (9%)

Percentages are rounded

*SD* standard deviation, *ISS* Injury Severity Score

^a^Based on the trauma level criteria according to the Dutch Trauma Society (NVT)

**Fig. 1:**
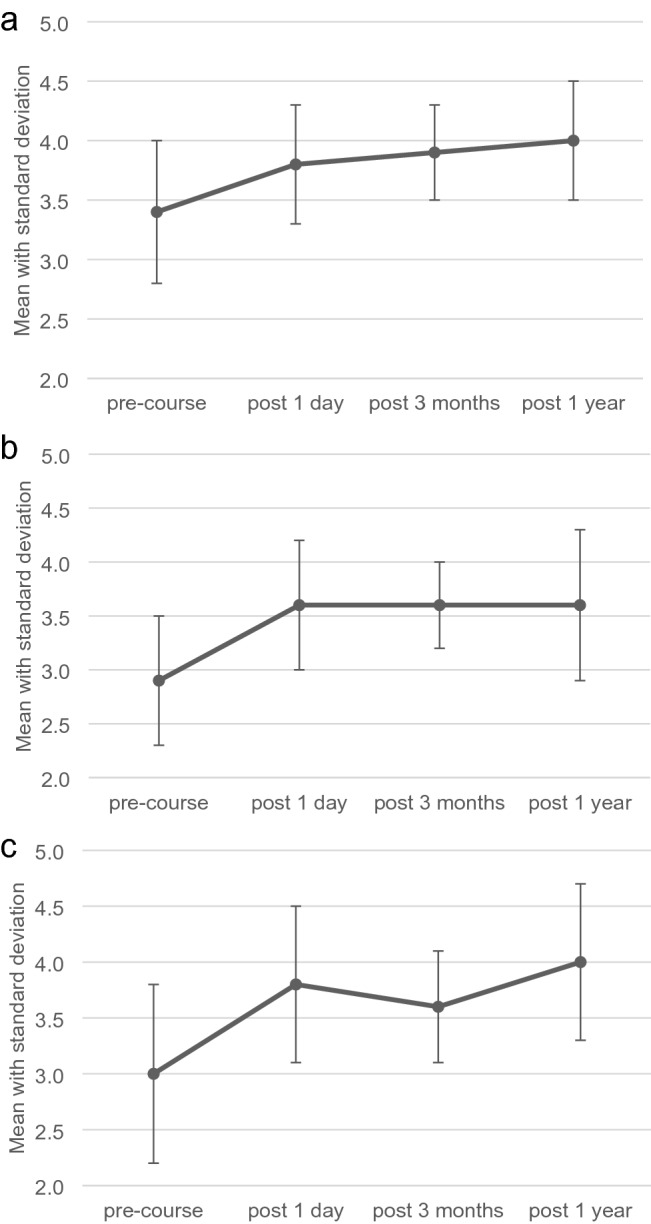
**a** Pooled ratings of nontechnical skills. **b** Pooled rating of technical skills. **c** Pooled rating of bleeding management skills

Ratings of self-assessed skills are listed in Table 2. All nontechnical, technical, and bleeding management skills statistically significantly increased after the course. After three months and one year, postcourse ratings remained higher than the precourse ratings. Pooled ratings are also displayed in Fig. 1a–c.

**Table 2:** Self-assessed confidence of skills

Skill	Mean rating (SD)		*p* value ^a^ *n* = 40	Mean rating (SD)	
	Pre-course	Post-course (1 day)		Post-course (3 months) *n* = 24	Post-course (1 year) *n* = 10
Nontechnical skills					
Communication in a team	3.9 (SD 0.6)	4.2 (SD 0.6)	0.03	4.2 (SD 0.6)	4.4 (SD 0.7)
Structural approach to the patient	3.8 (SD 0.7)	4.2 (SD 0.5)	0.001	4.1 (SD 0.5)	4.3 (SD 0.5)
Assessing the injuries and prioritize	3.6 (SD 0.6)	3.8 (SD 0.6)	< 0.001	4.1 (SD 0.5)	4.3 (SD 0.5)
Handling mass casualty situations	2.4 (SD 0.9)	3.0 (SD 0.9)	< 0.001	3.4 (SD 0.8)	3.1 (SD 0.7)
Leadership in a team	3.5 (SD 0.8)	3.7 (SD 0.9)	0.005	3.7 (SD 0.8)	4.1 (SD 0.9)
Technical skills					
Traumatic injuries	3.6 (SD 0.7)	3.8 (SD 0.6)	0.01	4.0 (SD 0.4)	4.2 (SD 0.4)
Trauma patients with ISS ≥ 16	3.0 (SD 0.9)	3.4 (SD 0.9)	< 0.001	3.5 (SD 0.8)	3.6 (SD 0.7)
Penetrating injuries	2.5 (SD 1.0)	3.4 (SD 0.9)	< 0.001	3.4 (SD 0.8)	3.2 (SD 0.9)
Blast injuries	2.0 (SD 0.9)	3.0 (SD 0.9)	< 0.001	2.9 (SD 0.8)	2.7 (SD 0.9)
Performing surgical airway	2.6 (SD 1.0)	3.7 (SD 0.9)	< 0.001	3.4 (SD 0.8)	3.4 (SD 1.0)
Head and neck injuries	2.8 (SD 1.0)	3.6 (SD 0.8)	< 0.001	3.6 (SD 0.7)	3.3 (SD 1.2)
Thoracic injuries	2.9 (SD 0.9)	3.7 (SD 0.8)	< 0.001	3.7 (SD 0.6)	3.5 (SD 1.1)
Abdominal injuries	2.9 (SD 1.0)	3.7 (SD 0.8)	< 0.001	3.6 (DS 0.8)	3.4 (SD 1.2)
Pelvic injuries	3.2 (SD 0.7)	3.7 (SD 0.8)	< 0.001	3.9 (SD 0.7)	4.0 (SD 0.5)
Extremity injuries	3.9 (SD 0.6)	4.1 (SD 0.8)	0.02	4.2 (SD 0.7)	4.0 (SD 0.5)
Extremes of age (< 16 or > 75)	3.0 (SD 0.9)	3.5 (SD 0.9)	< 0.001	3.6 (SD 0.7)	3.5 (SD 0.7)
Understanding of anatomy	N/A	4.2 (SD 0.6)	N/A	4.0 (SD 0.6)	4.3 (SD 0.5)
Bleeding management skills					
Bleeding control	3.1 (SD 0.7)	4.0 (SD 0.5)	< 0.001	3.7 (SD 0.6)	4.0 (SD 0.7)
Managing shock	3.3 (SD 0.9)	3.9 (SD 0.9)	< 0.001	3.8 (SD 0.6)	4.2 (SD 0.8)
Managing massive blood transfusion	3.0 (SD 0.9)	3.8 (SD 0.9)	< 0.001	3.7 (SD 0.8)	4.1 (SD 0.9)
Managing coagulopathy	2.7 (SD 1.0)	3.4 (SD 0.9)	< 0.001	3.3 (SD 0.7)	3.7 (SD 1.1)
Pooled ratings					
Pooled nontechnical skills	3.4 (SD 0.6)	3.8 (SD 0.5)	< 0.001	3.9 (SD 0.4)	4.0 (SD 0.5)
Pooled technical skills	2.9 (SD 0.6)	3.6 (SD 0.6)	< 0.001	3.6 (SD 0.4)	3.6 (SD 0.7)
Pooled bleeding management skills	3.0 (SD 0.8)	3.8 (SD 0.7)	< 0.001	3.6 (SD 0.5)	4.0 (SD 0.7)

Scale: 1 fully incompetent – 5 fully competent

^a^Comparison between precourse and 1-day postcourse rating by use of Wilcoxon signed rank test (*n* = 40 after exclusion of unpaired responses). Results from the follow-up questionnaire after three months and one year were not included in the comparative analyses due to the low absolute number of respondents

### Semi-structured interviews

Eleven DSATC course participants from three different specialties (trauma surgery, orthopedic surgery, and anesthesiology) participated in the interviews (Table 3). The themes identified from participants’ answers were linked to three main factors associated with changes in technical and nontechnical skills and their implementation in novel work situations: personal context, learning context, and skill transfer to work context.

**Table 3:** Background characteristics of interviewees

Characteristics	Value (*n* = 11)
Gender (*n*)	
Male	9
Female	2
Specialty (*n*)	
Trauma surgery	5
Orthopedic surgery	3
Anesthesiology	3
Work experience (*n*)	
Resident	3
Newly certified attending	3
Attending	5
Years of experience as attending (mean, SD)	6.9 (SD 3.9)
Hospital type (*n*)	
Academic	6
Peripheral	5

### Personal context

#### High starting levels limit gains in domain-specific knowledge and technical skills

Although participants with a background in anesthesiology indicated they gained several surgical skills (e.g., chest tube placement or performing a thoracotomy), most participants did not experience a substantial increase in their domain-specific knowledge and skills. More experienced participants stated that the course primarily refreshed their memory on how to react during certain circumstances, particularly situations with uncommon trauma pathology or pathology requiring rarely performed interventions (Quote 1).

*Quote 1 “It is always more of a refresher course. It is nice to practice some maneuvers that are not often required in daily practice.” (R10)*

### Learning context

#### Active exploratory learning in a realistic, controlled environment

The course design was generally considered a good mixture of theoretical and practical sessions. Almost all participants agreed that the hands-on workshops were the most educational and memorable parts. In particular, workshops in the operative porcine laboratory were deemed invaluable. The main reasons participants reported for this were that the simulations were realistic and that theory was integrated with practical skills (Quote 2). One participant mentioned that learning efficiency was high during the practical sessions because he was taken out of his comfort zone.

*Quote 2 “The alternation between the theoretical, anatomical and the actual cutting aspects are very valuable. […] To have to find a solution [to an injury] is a brilliant way to learn, because there is a coach to help you and answer your questions in a controlled environment.” (R10).*

#### Multidisciplinary learning: Insight into colleagues' perspectives

Participants agreed that the multidisciplinary aspect of the course was unique and very valuable because it resembles the real work environment and provides the opportunity to learn from other trauma team members’ perspectives. Especially during multidisciplinary case discussions in small groups, various specialties provided insight into different approaches (Quote 3).

*Quote 3 “The discussion panels are interesting because the gray areas in the different specialisms come to light. Different considerations around what, why and when the practitioner decides on a treatment are very valuable.” (R11).*

#### Supervisors’ expertise in advanced trauma care

Input by supervisors was deemed relevant by most participants (Quote 4). It was reasoned that the quality of case discussions depended on the supervisor’s experience. Some participants mentioned that the international faculty had considerable experience in injuries infrequently encountered in the Netherlands and provided useful tips to deal with these situations. However, two participants estimated that they had the same experience level as their supervisor during the course, making them question the additional value of their supervisor’s input.

*Quote 4 “I thought it was a good mixed group, with supervisors who could bring depth to the subjects and who bring something extra because they master the knowledge well.” (R7)*

### Transfer to work context

#### Increased self-efficacy and skill application in novel work situations

All but one participant agreed that course participation gave them more confidence in their trauma management skills. It was elaborated (Quote 5) that the course facilitated this gain in confidence by the application of newly acquired knowledge and skills to various complex cases of hemodynamically unstable patients in a controlled environment during discussions and hands-on workshops. Participants also reported an increased ability to correctly apply damage control principles after course participation. Other results mentioned by one participant were a greater ability to remain calm when providing trauma care and to take leadership.

*Quote 5 “It [the porcine laboratory] is just brilliant: trying to solve a real injury in a controlled environment in which you are coached and able to ask questions. That is not possible with real patients at work.” (R10).*

Furthermore, participants mentioned a broader application of domain-specific knowledge and skills in various novel work situations (Quote 6). One participant declared greater engagement in informal multidisciplinary discussions more frequently at the workplace (Quote 7).

*Quote 6*

*R: “For example, shortly after the course, [there was a need for a] surgical airway, but initially we did not manage to find someone to perform it and eventually we [the anesthesiologists] performed it ourselves, because we were the most experienced at that time. […] Multiple trauma cases arrived at the same time and I suggested to start, without a surgeon, with the initial assessment and basic management. […]”.*

*I: “Were these situations and cases similar to those practiced during the course?”*

*R: “No, not at all.” (R1)*

*Quote 7 “I ask colleagues from other specialties more frequently about their considerations [regarding patient care] to improve my knowledge.” (R7)*

#### Broadening reflective practice

All but one participant declared that they frequently, often daily, reflect on their performance and learning in the workplace both individually and with colleagues or supervisors. Reflection could occur during or immediately after a situation or after working hours (Quote 8).

*Quote 8 “[When I reflect on my own performance] I often do it by reviewing the situation again in my head. What also contributes [to my reflection] is reviewing the situation with colleagues and asking for advice about what I could have done differently. Sometimes it is hard to make a change if you stay within your own circle of thought.” (R2)*.

According to most participants, participation in the DSATC course did not change their frequency of reflection, but for four participants it broadened their views on which aspects to include. The course helped them gain a helicopter view and assess and reflect on the overall picture instead of solely on their individual knowledge and skills (Quote 9).

*Quote 9*

*I: “Did the quantity or way of your reflection change after course participation?”*

*R: “I do not think the quantity [has changed], but the way of reflecting as I have gained more of a helicopter view.” (R5)*

## Discussion

Trauma care providers reported increased overall self-efficacy in trauma skills after DSATC course participation but mentioned no substantial improvements in domain-specific knowledge and skills. The greatest advancements experienced in the work environment resulted from enhanced self-efficacy. The qualitative analyses revealed that the DSATC course empowered a broader application of participants’ knowledge and skills in novel work situations. The innovative application of skills and a broadened view of reflection on work processes and performance indicates the development of adaptive expertise. Workshops in the operative porcine laboratory, small-group case discussions, integrated multidisciplinary training, and highly experienced faculty members were regarded as course features that predominantly contributed to these learning results and improved the multidisciplinary teamwork.

An increase in self-efficacy in trauma management skills after the trauma masterclass participation is in line with the preceding research among participants of the DSATC courses between 2013 and 2016, the ASSET, ATOM, and Norwegian trauma course [8–10, 12]. Remarkably, Ali et al. found that knowledge on trauma management measured on a multiple-choice test did not improve as much as expected after the ATLS course [9]. Along with this finding, our study participants mentioned that domain-specific knowledge did not significantly increase with course participation. This outcome suggests that the greatest benefits of these trauma courses lie within increased self-efficacy and nontechnical skills rather than domain-specific knowledge and skills.

Note that the quantitative data on self-efficacy within this study represents a group of different medical professions with potentially divergent skill sets to begin with. Previous pre-post comparisons of self-efficacy per profession did not show significant differences between surgeons and anesthesiologists, and their postcourse gain in skills lasted for 2 years [8]. Self-efficacy ratings of scrub and anesthesia nurses were included in the current research to assess which effect course participation has on trauma skills of the trauma team as a whole. However, the semi-structured interviews only focused on the work perspectives of specialists in surgery and anesthesiology, because they made up the majority of course participants. For further strengthening of the whole trauma team, future projects should focus on the postcourse experienced changes in technical and nontechnical skills applied at work by scrub and anesthesia nurses.

Identifying and analyzing the DSATC course features that contribute most to participants’ nontechnical skills facilitates the extrapolation of the study findings, resulting in suggestions for future trauma curricula. First, simulation-based multidisciplinary team training should be more widely incorporated in trauma and emergency care masterclasses because it improves team-based skills when followed by feedback and reflection [26, 27]. The multidisciplinary case-based workshops in the DSATC anatomy and operative porcine laboratories closely simulate real working conditions, minimizing the consequences of learners’ actions compared to learning in the workplace. According to Hatano and Inagaki, such an environment stimulates adaptive expertise because learners gain the opportunity to take risks by experimenting with the application of their skills and knowledge in novel ways [15]. Second, high process variability (i.e., practicing with varying patient cases in multiple environments), complexity, and difficulty stimulate learners to apply their knowledge flexibly to varying situations [15, 16, 21, 28].

Finally, supervisors hold a significant role in the development of adaptive expertise. DSATC supervisors facilitated error-based learning during the course by debriefing with the team after each case in the laboratory workshops. Learning through errors seems to improve the adaptive transfer of expertise provided that learners were informed of how their errors related to the knowledge and skills being practiced [15, 29, 30]. Proper debriefing alone can enhance individual and team performance by 20–25% [31]. Additionally, a working climate in which supervisors indicate they value and support their employees contributes to higher levels of adaptive expertise [32]. Having a supervisor’s trust allows greater experimentation in a learner’s approach to novel situations at work. Awareness of the supervisor’s vital position is important, and participation in teach-the-teacher training is recommended for future trauma masterclass organizations.

Self-efficacy and adaptive expertise are fundamental qualities for the adequate functioning of trauma care providers but additionally facilitate the transfer of acquired skills to the work environment by promoting experimentation and practice of skills in the workplace. In line with the social cognitive theory [7], medical professionals who experience increased self-efficacy are more likely to implement innovations in work situations they previously would not have had the confidence to implement.

A remaining issue is the limited exposure to complex trauma cases in the workplace. Still, lower exposure is not necessarily related to the trauma level of the hospital where participants are employed. Exposure to severely injured polytrauma patients might also be limited in academic level 1 trauma centres, due to a geographically defined low incidence of penetrating injuries [33], and because of a generally greater staffing which limits individual learning opportunities. Other solutions have to be established to facilitate adequate exposure to complex trauma injuries and enhance retainment of damage control skills. Therefore, in the Netherlands, Van der Wal et al. initiated a civilian-military collaborative program in trauma training between the Ministry of Defense and the University of KwaZulu-Natal in South Africa [34]. This mutually beneficial program could, among other things, provide substantial trauma exposure within short periods.

### Strengths and limitations

This study on the effectiveness of a trauma masterclass is the first to include a description of teaching formats’ success factors in the context of learning theories. The mixed methods design produces quantitative self-efficacy ratings that can be easily compared with existing or future literature. Additionally, this information is supplemented by a qualitatively reported deeper exploration of participants’ perceptions of how and why certain teaching formats produce results that are transferred to the workplace. Data gathered with the interviews reflect a significant follow-up period of 9 months, and was derived with achievement of data saturation. Other trauma course organizations can adopt the teaching formats that this study identified as effective to enhance technical and nontechnical skills.

Unfortunately, there was a significant loss to follow-up on postcourse questionnaires which resulted in a relatively small study population within the survey. Another limitation regarding follow-up measurements within the survey is that self-assessed trauma skills are possibly influenced by progressing work experience or formal learning activities. On the contrary, a study that assessed trauma skills 2 years after the ATLS course found a persistent, significant difference in objectively assessed clinical performance between two groups that attended different course formats [35]. This outcome suggests there might be an independent correlation with the course working formats, even after consecutive work experience.

Technical and nontechnical skills were subjectively evaluated in this study, which should be considered while interpreting the study results. The extent to which self-assessment reflects actual skills was not objectively assessed because this did not fall under the study aim.

Last, no validated measurement instrument was available to assess adaptive expertise in healthcare workers. Future research is required to develop such a tool, and promising projects are currently being rolled out by a Dutch research collaboration titled Adapt at Work [36]. Another interesting focus for further research would be to study the impact of similar continuing education courses on patient outcomes, although development of an appropriate research methodology can be challenging due to the many additional influences on patient outcomes.

## Conclusions

Through enhanced overall self-efficacy in trauma skills, improved reflection methods, and strengthened multidisciplinary teamwork, the DSATC course format provides participants with the required tools to handle unexpected and challenging situations during trauma care. A paradigm shift from trauma team training primarily focusing on domain-specific skills to training on a more overarching level could contribute to increased self-efficacy, adaptive expertise, and multidisciplinary teamwork. Adding this as an explicit focus of future trauma curricula is essential to prepare trauma teams for the highly variable and complex situations they face at work.

## Supplementary Information

Below is the link to the electronic supplementary material.

Supplementary file1 (PDF 123 KB)

Supplementary file2 (PDF 66 KB)

Supplementary file3 (PDF 83 KB)

Supplementary file4 (PDF 104 KB)

## Data Availability

The quantitative datasets from the self-efficacy questionnaires are available from the corresponding author on reasonable request. Raw data from the interviews will not be shared due to the potential traceability to the individual.
