# Supplementary material for: Self-efficacy and application of skills in the workplace after multidisciplinary trauma masterclass participation: a mixed methods survey and interview study
Source: Eur J Trauma Emerg Surg. 2022 Nov 10;49(2):1101–11. doi: 10.1007/s00068-022-02159-8 (PMC9647757; doi:10.1007/s00068-022-02159-8)
Supplement: Supplementary file 1 — Supplementary file1 (PDF 123 KB) [file 68_2022_2159_MOESM1_ESM.pdf]

Online Resource 1. DSATC self-efficacy questionnaire

Title

Self-efficacy and application of skills in the workplace after multidisciplinary trauma masterclass participation - A mixed methods survey and interview study

Journal

European Journal of Trauma and Emergency Surgery

Authors

Frederike J.C. Haverkamp, Idris Rahim, Rigo Hoencamp, Cornelia R.M.G. Fluit, Kees J.H.M. van Laarhoven, Edward C.T.H. Tan

Corresponding author

Frederike J.C. Haverkamp, MD

Department of Surgery, Radboudumc, Nijmegen, the Netherlands

E-mail: Frederike.haverkamp@radboudumc.nl

QUESTIONNAIRE

	**Characteristics of participants**	
1	Gender	Male | Female
2	Age	Years
3	Background	Surgery | Anesthesiology | OR nurse
4	Specialty	Surgeon | Orthopedic surgeon | Anesthesiologist | Resident | Fellow
5a	Time in training as a resident	Years
5b	Time as a consultant	Years
6	Faculty member	Yes | No
7	Type of hospital currently employed	Level 1 trauma center | Level 2 trauma center | Level 3 trauma center | Other (specify)
8	Military experience (if yes: 8a and 8b)	Yes | No
8a	What was the location?	
8b	How many months have you worked there?	
9	Experience working in austere environment (if yes: 9a and 9b)	Yes | No
9a	What was the location?	
9b	How many months have you worked there?	
10	Experience in trauma care	Years
11	Number of patients treated with ISS >15 each year	0 | 1-4 | 5-9 | 10-14 | >15
12	Number of patients treated with penetrating injury each year	0 | 1-4 | 5-9 | 10-14 | >15
13	Number of patients treated with damage control surgery	0 | 1-4 | 5-9 | 10-14 | >15

	Self-assessed confidence in performing skills					
		1[$]	2	3	4	5[$]
	**Non-technical skills**					
1	Communication in a team	O	O	O	O	O
2	Structural approach to the patient	O	O	O	O	O
3	Assessing the injuries and prioritize	O	O	O	O	O
4	Handling mass casualty situations	O	O	O	O	O
5	Leadership in a team	O	O	O	O	O
	**Technical skills**					
6	Treatment of patients with traumatic injuries	O	O	O	O	O
7	Treatment of trauma patients with ISS>16	O	O	O	O	O
8	Treatment of patients with penetrating injuries	O	O	O	O	O
9	Treatment of patients with blast injuries	O	O	O	O	O
10	Performing surgical airway	O	O	O	O	O
11	Recognition and treatment of head/neck injuries	O	O	O	O	O
12	Recognition and treatment of thorax injuries	O	O	O	O	O
13	Recognition and treatment abdominal injuries	O	O	O	O	O
14	Recognition and treatment of pelvic injuries	O	O	O	O	O
15	Recognition and treatment of extremity injuries	O	O	O	O	O
16	Resuscitation of patients with extremes of age (<16 or >75)	O	O	O	O	O
	**Oher skills**					
17	Bleeding control	O	O	O	O	O
18	Managing shock	O	O	O	O	O
19	Managing massive blood transfusion	O	O	O	O	O
20	Managing coagulopathy	O	O	O	O	O
	**Evaluation**					
21	I am satisfied with what I learned	O	O	O	O	O
22	Understanding of surgical anatomy	O	O	O	O	O

[$]1 fully incompetent – 5 fully competent
