# Supplementary material for: Self-efficacy and application of skills in the workplace after multidisciplinary trauma masterclass participation: a mixed methods survey and interview study
Source: Eur J Trauma Emerg Surg. 2022 Nov 10;49(2):1101–11. doi: 10.1007/s00068-022-02159-8 (PMC9647757; doi:10.1007/s00068-022-02159-8)
Supplement: Supplementary file 2 — Supplementary file2 (PDF 66 KB) [file 68_2022_2159_MOESM2_ESM.pdf]

Online Resource 2. Interview guide

Title

Self-efficacy and application of skills in the workplace after multidisciplinary trauma masterclass participation - A mixed methods survey and interview study

Journal

European Journal of Trauma and Emergency Surgery

Authors

Frederike J.C. Haverkamp, Idris Rahim, Rigo Hoencamp, Cornelia R.M.G. Fluit, Kees J.H.M. van Laarhoven, Edward C.T.H. Tan

Corresponding author

Frederike J.C. Haverkamp, MD

Department of Surgery, Radboudumc, Nijmegen, the Netherlands

E-mail: Frederike.haverkamp@radboudumc.nl

INTERVIEW GUIDE

Effect on technical and non-technical skills

- What did you learn from the course?
  - Why do you think you specifically remember this?
  - Why do you think you do not remember anything specific? How could the course be improved for better retainment of knowledge?
- What has been the influence of DSATC course participation on your work performance?
- Have you applied the acquired skills at work?
  - Can you describe the situation and how you applied the skills acquired during the DSATC course?
- Have situations occurred in which you were uncertain about your own performance and skills? Did this change after the DSATC course?
- In which way do you keep your domain specific knowledge up to date? Did this change after course participation?
- Did you reflect on your performance during the course?
- (How) do you reflect on your performance in the workplace? Did this change after course participation? What do you think has caused this change?
- When do you think, you will need to take a refresher course? Why?

Learning activities

- What was the most memorable part of the course for you? Why?
- Which part of the course had the most educational value for you? Why?
- What is your opinion about the preparation material for the course?
- What is your opinion about the content and structure of the physical course days?

- What is your opinion about course duration?
    - In case the course would be shortened, in which theoretical/practical ratio should this be done?
- What is your opinion about the multidisciplinary aspect of the course?
- Did you get enough opportunities to practice with unfamiliar situations during the course?
- Do you have any other remarks?
