# Supplementary material for: Self-efficacy and application of skills in the workplace after multidisciplinary trauma masterclass participation: a mixed methods survey and interview study
Source: Eur J Trauma Emerg Surg. 2022 Nov 10;49(2):1101–11. doi: 10.1007/s00068-022-02159-8 (PMC9647757; doi:10.1007/s00068-022-02159-8)
Supplement: Supplementary file 3 — Supplementary file3 (PDF 83 KB) [file 68_2022_2159_MOESM3_ESM.pdf]

Online Resource 3. Reflexivity statement

Title

Self-efficacy and application of skills in the workplace after multidisciplinary trauma masterclass participation - A mixed methods survey and interview study

Journal

European Journal of Trauma and Emergency Surgery

Authors

Frederike J.C. Haverkamp, Idris Rahim, Rigo Hoencamp, Cornelia R.M.G. Fluit, Kees J.H.M. van Laarhoven, Edward C.T.H. Tan

Corresponding author

Frederike J.C. Haverkamp, MD

Department of Surgery, Radboudumc, Nijmegen, the Netherlands

E-mail: Frederike.haverkamp@radboudumc.nl

REFLEXIVITY STATEMENT

With regard to the qualitative interviews, researchers' backgrounds were strategically allocated among research activities to include various perspectives and thereby optimally explore the collected data. Development of the interview guide took place with input from F.H., I.R., C.F., and E.T., including perspectives from outsiders of the DSATC course (F.H. and I.R.), an educational specialist (C.F.), and a former DSATC course director with an extensive background in surgery and emergency medicine education (E.T.). Interviews were performed by a DSATC course outsider (I.R.) to limit bias from socially desirable answers. Data analysis and interpretation was performed from insider- (R.H., E.T.), outsider- (F.H., I.R.) and educational perspectives (LCF., C.v.L., E.T.), to ensure an open approach while simultaneously being able to place findings into the relevant context. All researchers, however, are expected to have been sensitized to statements related to adaptive expertise.
