# Supplementary material for: Self-efficacy and application of skills in the workplace after multidisciplinary trauma masterclass participation: a mixed methods survey and interview study
Source: Eur J Trauma Emerg Surg. 2022 Nov 10;49(2):1101–11. doi: 10.1007/s00068-022-02159-8 (PMC9647757; doi:10.1007/s00068-022-02159-8)
Supplement: Supplementary file 4 — Supplementary file4 (PDF 104 KB) [file 68_2022_2159_MOESM4_ESM.pdf]

Online Resource 4. Characteristics of dropouts

Title

Self-efficacy and application of skills in the workplace after multidisciplinary trauma masterclass participation - A mixed methods survey and interview study

Journal

European Journal of Trauma and Emergency Surgery

Authors

Frederike J.C. Haverkamp, Idris Rahim, Rigo Hoencamp, Cornelia R.M.G. Fluit, Kees J.H.M. van Laarhoven, Edward C.T.H. Tan

Corresponding author

Frederike J.C. Haverkamp, MD

Department of Surgery, Radboudumc, Nijmegen, the Netherlands

E-mail: Frederike.haverkamp@radboudumc.nl

CHARACTERISTICS OF DROPOUTS

	Value (n=8)
Sex (n)	
Male	3
Female	5
Age (mean, SD)	36.9 (SD 5.9)
Profession (n)	
Surgeon	5
Scrub nurse	1
Anesthesiologist	2
Trauma center level [a] of current worksite (n)	
Level 1	5
Level 2	2
Level 3	1
Years of experience in trauma care (mean, SD)	5.8 (SD 4.3)
Military experience (n)	
Yes	1
No	7
Experience working in austere environment (n)	
Yes	1
No	7
Number of patients treated each year with traumatic injury (n)	
0	0
1-4	1
5-9	0
10-14	0
≥15	7
Number of patients treated each year with ISS >15 (n)	
0	1
1-4	0
5-9	2
10-14	2
≥15	3
Number of patients treated each year with penetrating trauma (n)	
0	2
1-4	5
5-9	1
10-14	0
≥15	0
Number of patients treated each year requiring damage control surgery (n)	
0	2
1-4	4
5-9	1
10-14	0
≥15	1

[a] *Based on the trauma level criteria according to the Dutch Trauma Society (NVT)*

SD Standard Deviation; ISS Injury Severity Score
